# Supplementary material for: Knowledge, attitudes, and practices against the growing threat of COVID-19 among medical students of Pakistan
Source: PLoS One. 2020 Dec 11;15(12):e0243696. doi: 10.1371/journal.pone.0243696 (PMC7732088; doi:10.1371/journal.pone.0243696)
Supplement: Attachment — Submitted filename: Rebuttal letter doc (1).docx [file pone.0243696.s002.docx]

PONE-D-20-23756
Knowledge, Attitudes, and practices against the growing threat of COVID-19 among medical students of Pakistan
PLOS ONE

Dear editor,

Pls find the modified manuscript. We have incorporated all suggestions recommended by the reviewers’. Following is the point-wise reply to the reviewers' comments.

With best regards,

Dr Noreen

**Reply to comments**

A rebuttal letter that responds to each point raised by the academic editor and reviewer(s). You should upload this letter as a separate file labeled 'Response to Reviewers'.

**Reply:** Rebuttal letter is being uploaded as a separate file.

A marked-up copy of your manuscript that highlights changes made to the original version. You should upload this as a separate file labeled 'Revised Manuscript with Track Changes'.

**Reply:** The revised manuscript is being uploaded as a separate file as

an unmarked version of your revised paper without tracked changes.

You should upload this as a separate file labeled 'Manuscript'.

**Reply:** Unmarked manuscript is being uploaded as a separate file.

1. Please ensure that your manuscript meets PLOS ONE's style requirements, including those for file naming. The PLOS ONE style templates can be found at

https://journals.plos.org/plosone/s/file?id=wjVg/PLOSOne_formatting_sample_main_body.pdf and

https://journals.plos.org/plosone/s/file?id=ba62/PLOSOne_formatting_sample_title_authors_affiliations.pdf

**Reply:** We have modified the style of our manuscript as required by the Plos One.

2. We note that you have indicated that data from this study are available upon request. PLOS only allows data to be available upon request if there are legal or ethical restrictions on sharing data publicly. For information on unacceptable data access restrictions, please see http://journals.plos.org/plosone/s/data-availability#loc-unacceptable-data-access-restrictions.

**Reply:**

In your revised cover letter, please address the following prompts:

a) If there are ethical or legal restrictions on sharing a de-identified data set, please explain them in detail (e.g., data contain potentially identifying or sensitive patient information) and who has imposed them (e.g., an ethics committee). Please also provide contact information for a data access committee, ethics committee, or other institutional body to which data requests may be sent.

 b) If there are no restrictions, please upload the minimal anonymized data set necessary to replicate your study findings as either Supporting Information files or to a stable, public repository and provide us with the relevant URLs, DOIs, or accession numbers. Please see http://www.bmj.com/content/340/bmj.c181.long for guidelines on how to de-identify and prepare clinical data for publication. For a list of acceptable repositories, please see <http://journals.plos.org/plosone/s/data-availability#loc-recommended-repositories>.

We will update your Data Availability statement on your behalf to reflect the information you provide.

**Reply:** SPSS data sheet is being uploaded.

3. Please ensure that you include a title page within your main document. You should list all authors and all affiliations as per our author instructions and clearly indicate the corresponding author.

**Reply:** The title page has been included in the main document.

4. Please remove your figures from within your manuscript file, leaving only the individual TIFF/EPS image files, uploaded separately.  These will be automatically included in the reviewers’ PDF.

**Reply:** Figures have been separated and have been converted to TIFF.

Reviewers' comments:

Reviewer's Responses to Questions

**Comments to the Author**

1. Is the manuscript technically sound, and do the data support the conclusions?

The manuscript must describe a technically sound piece of scientific research with data that supports the conclusions. Experiments must have been conducted rigorously, with appropriate controls, replication, and sample sizes. The conclusions must be drawn appropriately based on the data presented.

Reviewer #1: Partly

Reviewer #2: Yes

**Reply:** Thank you for your comments.

2. Has the statistical analysis been performed appropriately and rigorously?

Reviewer #1: No

Reviewer #2: Yes

**Reply:** We have included 95% CI with OR in table 4, as suggested by the reviewer.

3. Have the authors made all data underlying the findings in their manuscript fully available?

The [PLOS Data policy](http://www.plosone.org/static/policies.action#sharing) requires authors to make all data underlying the findings described in their manuscript fully available without restriction, with rare exception (please refer to the Data Availability Statement in the manuscript PDF file). The data should be provided as part of the manuscript or its supporting information, or deposited to a public repository. For example, in addition to summary statistics, the data points behind means, medians and variance measures should be available. If there are restrictions on publicly sharing data—e.g. participant privacy or use of data from a third party—those must be specified.

Reviewer #1: Yes

Reviewer #2: Yes

**Reply:** Thank you for your comments.

4. Is the manuscript presented in an intelligible fashion and written in standard English?

PLOS ONE does not copyedit accepted manuscripts, so the language in submitted articles must be clear, correct, and unambiguous. Any typographical or grammatical errors should be corrected at revision, so please note any specific errors here.

Reviewer #1: Yes

Reviewer #2: Yes

**Reply:** Thank you for your comments.

5. Review Comments to the Author

Please use the space provided to explain your answers to the questions above. You may also include additional comments for the author, including concerns about dual publication, research ethics, or publication ethics. (Please upload your review as an attachment if it exceeds 20,000 characters)

Reviewer #1: Good effort by the investigators. However, there are some major concerns with the methods and analyses that need to be addressed.
1. How were the main outcomes of knowledge and practice adequacy defined? Please provide rationales for choosing the cut-off points for positive attitudes and adequate practice.

**Reply:** Actually, these are very subjective terms. However, it was considered that if a person has > 70% of the correct knowledge of COVID-19 related questions, then it was considered adequate. In case of attitude, if a person was saying “No” or “not sure” for questions such as “It is my social responsibility to take safety measures in controlling the spread of this infection." It means he/she has a negative attitude, and for four attitude questions, the total score ranged from -4 to +4. The plus scores were taken as positive attitudes, while negative scoring indicated a negative attitude.

Similarly, in the practice section, there were six questions. For practice questions, 2 points were awarded for yes, one for sometimes and zero for no, and the score =>6 scores was taken as adequate and < 6, was taken as inadequate."

2.What was the response rate? How many medical students were invited to participate vs those who participated in the survey? This has implications on the generalizability of findings to within Pakistan.

**Reply:** We sent an invitation to different medical universities. A total of 1800 medical students were approached, complete responses were acquired from 1474 medical students with a response rate of 82%. This has been included in the methods.

3.Please provide 95% CI for the OR in Table 4. As mentioned above, please clarify whether the models have adjusted for any variables.

**Reply:** In table 4, 95% CI for the OR has been added. The model was not adjusted for any variables.

4. How do the findings about factors associated with COVID-19 related knowledge and practice adequacy in Pakistan compare with literature from elsewhere?

**Reply:**

5.In the discussion, please add implications of the findings, and what steps are needed to address the gaps identified?

**Reply:**

Other comments:

Please proofread and correct grammatical errors throughout the paper.

**Reply:** A professional editing service has done the language editing and a certificate is attached.

Please make the format of the paper consistent (font, color, line spaces) and provide the complete tables.

**Reply:** The manuscript has been formatted, and tables have been completed as suggested.

**Reviewer #2:**

This is an interesting paper and is written at time when it is needed. Furthermore, this manuscript is written on issue which is very relevant in todays world. I have no comment to make hence ask for acceptance.

Reply:

Thank you very much for liking our manuscript.
