# Supplementary material for: Knowledge, attitudes, and practices against the growing threat of COVID-19 among medical students of Pakistan
Source: PLoS One. 2020 Dec 11;15(12):e0243696. doi: 10.1371/journal.pone.0243696 (PMC7732088; doi:10.1371/journal.pone.0243696)
Supplement: Attachment — Submitted filename: 2ND REVISION Response to reviewer.docx [file pone.0243696.s003.docx]

Dear editor,

We are thankful to the worthy reviewers for sparing their valuable time to review our manuscript. Please find the description and justification of point-to-pint comments and line numbers. Kindly note that numbering in the document is not continuous because of section breaks.

With best regards,

Dr Khola

| **Reviewer** | **Comment** | **Response** | **Reply/Changes** | |
| --- | --- | --- | --- | --- |
| **Review Comments to the Author** | | | | |
| **Reviewer** | **Comment** | **Response** | | **Changes** |
| 1 | Good effort by the investigators. However, there are some major concerns with the methods and analyses that need to be addressed. 1. How were the main outcomes of knowledge and practice adequacy defined? Please provide rationales for choosing the cut-off points for positive attitudes and adequate practice? | Actually, these are very subjective terms.  However, it was considered that if a person has > 70% of the correct knowledge of COVID-19 related questions, then it was considered adequate. In case of attitude, if a person was saying “No” or “not sure” for questions such as “It is my social responsibility to take safety measures in controlling the spread of this infection." It means he/she has a negative attitude, and for four attitude questions, the total score ranged from -4 to +4. The plus scores were taken as positive attitudes, while negative scoring indicated a negative attitude.  Similarly, in the practice section, there were six questions. For practice questions, 2 points were awarded for yes, one for sometimes and zero for no, and the score =>6 scores was taken as adequate and < 6, was taken as inadequate." | | Clean manuscript: Page 6&7  Line 147-158  Track changes:  Page 158-171 |
| 1 | What was the response rate? How many medical students were invited to participate vs those who participated in the survey? This has implications on the generalizability of findings to within Pakistan. | We sent an invitation to different medical universities. A total of 1800 medical students were approached, complete responses were acquired from 1474 medical students with a response rate of 82%. This has been included in the methods. | | Clean manuscript: Page 6  Line 142-145  Track changes:  Page 7  Line 152-155 |
| 1 | Please provide 95% CI for the OR in Table 4. As mentioned above, please clarify whether the models have adjusted for any variables. | In table 4, 95% CI for the OR has been added. The model was not adjusted for any variables. | | Page 14 |
| 1 | How do the findings about factors associated with COVID-19 related knowledge and practice adequacy in Pakistan compare with literature from elsewhere? | The findings about factors associated with COVID-19 related knowledge and practice adequacy given in current study compare with literature published from Pakistan and other countries too.  A Pakistani cross-sectional study has documented good knowledge, positive attitude, and reasonable practices regarding COVID-19 infection in primary healthcare providers from three tertiary care hospitals.  Hussain I, Majeed A, Imran I, Ullah M, Hashmi FK, Saeed H, et al. Knowledge, attitude, and practices toward COVID-19 in primary healthcare providers: a Cross-Sectional Study from three tertiary Care Hospitals of Peshawar, Pakistan. J Community Health. 2020:1-9. doi: [10.1007/s10900-020-00879-9](https://dx.doi.org/10.1007%2Fs10900-020-00879-9).  In contrast to our results, a Pakistani study reported low knowledge scores among the general public, including students.  Tariq S, Tariq S, Baig M, Saeed M. Knowledge, awareness and practices regarding novel coronavirus among a sample of Pakistani population, a cross-sectional study. Disaster Med Public Health Prep. 2020;1-20. doi:10.1017/dmp.2020.408  Research on medical students from Iran indicated a high level of related knowledge and high performance in preventive behaviours but moderate risk perception.  Taghrir MH, Borazjani R, Shiraly R. COVID-19 and Iranian medical students; a survey on their related-knowledge, preventive behaviors and risk perception. Arch Iran Med. 2020;23:249-254. doi:10.34172/aim.2020.06  In Jordan, medical and non-medical students obtained an average knowledge score. The majority of the participants had good knowledge of COVID-19 symptoms and were aware of the lack of vaccine and treatment for COVID-19.  Alzoubi H, Alnawaiseh N, Lubad MA, Aqel A, Al-Shagahin H. COVID-19 - knowledge, attitude and practice among medical and non-medical university students in Jordan. J Pure Appl Microbiol. 2020;14:17-24. doi:10.22207/JPAM.14.1.04 | | Clean manuscript: Page 16-17  Line 45-52  Track changes:  Page 17  Line 56-63 |
| 1 | In the discussion, please add Implications of the findings, and what steps are needed to address the gaps identified? | The following implications have been added. As a response to the COVID-19 pandemic majority of study participants embraced social isolation strategies, regular hand washing, and improved personal hygiene procedures as their initial line of defense against the COVID 19. Pakistani medical students presented a predictable level of knowledge about the COVID-19 and implemented appropriate strategies to stop its spread.  Moreover, dedicated training programs for medical professionals at the Government level can supplement their knowledge of risks and preventive strategies related to COVID-19, which will help them deliver proper care to their patients and keep themselves safe from the virus. Recently, the WHO Director-General's opening remarks at the media briefing on COVID-19 acclaimed Pakistan in deploying the infrastructure built up over many years for polio to combat COVID-19. Community health workers who have been trained to go door-to-door vaccinating children for polio have been utilized for surveillance, contact tracing, and care. Optimistically, by scheming effective COVID-19 prevention and management programs at the Government level, countries like Pakistan can manage the spread of COVID-19 infection. A worldwide public health driven strategy to improve knowledge should be conducted to combat the disease. In this regard, everyone should put efforts to eradicate this pandemic. | | Clean manuscript: Page 16& 17  Line 45-52  Track changes:  Page 18& 19  Line 88-109 |
|  | Please proofread and correct grammatical errors throughout the paper. | A professional editing service has done the language editing and a certificate is attached. | | Certificate is attached as “supporting information” |
|  | Please make the format of the paper consistent (font, color, line spaces) and provide the complete tables. | The manuscript has been formatted, and tables have been completed as suggested. | |  |
| 2 | This is an interesting paper and is written at time when it is needed. Furthermore, this manuscript is written on issue which is very relevant in todays world. I have no comment to make hence ask for acceptance. | Thank you very much for liking our manuscript. | |  |
